# A photophysiological model of coral bleaching under light and temperature stress: experimental assessment

**DOI:** 10.1093/conphys/coaf020

**Published:** 2025-04-15

**Authors:** Sophia L Ellis, Mark E Baird, Luke P Harrison, Kai G Schulz, Daniel P Harrison

**Affiliations:** National Marine Science Centre, School of Environment, Science and Engineering, Southern Cross University, Coffs Harbour, NSW 2450, Australia; Environment Research Unit, Commonwealth Scientific and Industrial Research Organisation, Hobart, TAS 7001, Australia; School of Aerospace, Mechanical and Mechatronic Engineering, University of Sydney, Sydney, NSW 2006, Australia; Centre for Coastal Biogeochemistry, School of Environment, Science and Engineering, Southern Cross University, Lismore, NSW 2480, Australia; National Marine Science Centre, School of Environment, Science and Engineering, Southern Cross University, Coffs Harbour, NSW 2450, Australia; School of Geosciences, University of Sydney, Sydney, NSW 2050, Australia

**Keywords:** Climate change, environmental stressor, irradiance, mechanistic, numerical model, oxidative stress, physiological model, Scleractinia

## Abstract

Marine heatwaves occurring against the backdrop of rising global sea surface temperatures have triggered mass coral bleaching and mortality. Irradiance is critical to coral growth but is also an implicating factor in photodamage, leading to the expulsion of symbiotic algae under increased temperatures. Numerical modelling is a valuable tool that can provide insight into the state of the symbiont photochemistry during coral bleaching events. However, very few numerical physiological models combine the influence of light and temperature for simulating coral bleaching. The coral bleaching model used was derived from the coral bleaching representation in the eReefs configuration of the CSIRO Environmental Modelling Suite, with the most significant change being the equation for the rate of detoxification of reactive oxygen species. Simulated physiological bleaching outcomes from the model were compared to photochemical bleaching proxies measured during an *ex situ* moderate degree-heating week (up to 4.4) experiment. The bleaching response of *Acropora divaricata* was assessed in an unshaded and 30% shade treatment. The model-simulated timing for the onset of bleaching under elevated temperatures closely corresponded with an initial photochemical decline as observed in the experiment. Increased bleaching severity under elevated temperature and unshaded light was also simulated by the model, an outcome confirmed in the experiment. This is the first experimental validation of a temperature-mediated, light-driven model of coral bleaching from the perspective of the symbiont. When forced by realistic environmental conditions, process-based mechanistic modelling could improve accuracy in predicting heterogeneous bleaching outcomes during contemporary marine heatwave events and future climate change scenarios. Mechanistic modelling will be invaluable in evaluating management interventions for deployment in coral reef environments.

## Introduction

Climate change is increasing the frequency and intensity of marine heatwaves (MHWs), which impact coral reefs globally. Anomalously high seawater temperatures associated with thermal stress events have resulted in a global decline in coral cover of >20% during this (21st) century (Berkelmans *et al.*, 2004; Hughes *et al.*, 2017; Hughes *et al.*, 2018). Thermal anomalies can lead to the breakdown of the mutualism between the coral host and their endosymbiotic algae (Glynn, 1993). Coral bleaching is characterised by a loss of pigmentation of algae from the family Symbiodiniaceae, an expulsion of Symbiodiniaceae via exocytosis from the coral host or the shedding of *in hospite* Symbiodiniaceae*-*containing host cells to the water column (Gates *et al.*, 1992).

Recent mass coral bleaching events on the Great Barrier Reef (GBR) have led to regional-scale changes in coral reef assemblages (Hughes *et al.*, 2018). The widespread events of 2016, 2017 and 2020 were more severe than those in preceding decades, with estimated coral loss across the entire GBR from these events ranging from 30 (Bozec *et al.*, 2022) to 50% in shallow waters after the 2016 event alone (Hughes *et al.*, 2017). Aerial surveys confirmed a fourth and fifth mass coral bleaching event impacted the GBR in 2022 and 2024 (Cantin *et al.*, 2024). During the last decade, the frequency of MHWs has increased by >50% (Oliver *et al.*, 2018; Méndez *et al.*, 2023). Using a 1982–2005 baseline, >50% of the world’s oceans are projected to be in a permanent MHW state by the end of the 21st century (Oliver *et al.*, 2019). Annual mass bleaching is predicted on >90% of coral reefs worldwide by the end of this century (Frieler *et al.*, 2013; van Hooidonk *et al.*, 2013).

A variety of modelling systems have been developed for projecting broadscale patterns of mass bleaching on decadal scales, incorporating various levels of complexity (Donner *et al.*, 2009). In a relatively simplistic approach, general circulation models (GCMs) forced by the IPCC IS92a emission scenario have been used to estimate the frequency of coral bleaching events using future predictions of sea surface temperatures (SSTs) combined with thermal thresholds (Hoegh-Guldberg, 1999). Bleaching severity at a broad scale is well correlated with the degree-heating week (DHW) stress metric (Sheppard, 1999; Spencer *et al.*, 2000). The DHW metric integrates anonymously high temperatures during the most recent 12-week period by summing any temperature anomaly at least 1°C above the maximum monthly mean (MMM) (Liu *et al.*, 2013).

Several stressors are now recognised to influence coral bleaching (Welle *et al.*, 2017). For instance, high irradiance increases the impacts of high temperatures on coral bleaching (Gleason and Wellington, 1993). The Light Stress Damage algorithm was developed to combine satellite-derived SST data and satellite-derived solar insolation data into a single measure of stress (Skirving *et al.*, 2017). Satellite algorithms do not consider bleaching factors that cannot be remotely sensed (Cantin *et al.*, 2021), namely dissolved nutrients (D’Angelo and Wiedenmann, 2014), ocean acidification, biological interactions and microbial communities. Modelling can overcome the limitations of satellite algorithms and better represent the variance in coral bleaching response under a range of dynamic environmental conditions.

Process-based models of the coral–symbiont relationship were developed to increase our understanding of climate change impacts on coral bleaching severity. Perhaps the most ambitious application of a coral physiological model to date is the coral bleaching model (CBM). Using extensions from previous models, the CBM was developed for a mechanistic description of the coral–symbiont relationship (Baird *et al.*, 2018). The CBM includes models for the coral polyp (Gustafsson *et al.*, 2013), photosystem bleaching (Gustafsson *et al.*, 2014), photoadaptation (Baird *et al.*, 2013) and multiple nutrient limitations of microalgae (Baird *et al.*, 2016). The CBM has been incorporated into the CSIRO Environmental Modelling Suite (Baird *et al.*, 2020b) and implemented in a ∼1-km resolution coupled hydrodynamic–biogeochemical model (a product of the eReefs Project; Steven *et al.*, 2019) that encompasses the entire length of the GBR (Baird *et al.*, 2020b). The CBM in the eReefs model has been calibrated against broadscale aerial bleaching surveys (Baird *et al.*, 2018; Baird *et al.*, 2021) and has captured the distribution and intensity of bleaching during the summers of 2016 (Baird *et al.*, 2018), 2017 and 2020 (Cantin *et al.*, 2021). The CBM applied in realistic environmental conditions has the potential to provide more detailed bleaching predictions than those available from satellite-based coral bleaching parameters (Steven *et al.*, 2019).

The eReefs marine biogeochemical (BGC) model simulates the environmental conditions of the GBR and is used in its management. Previous applications include assessing the impacts of catchment run-off on reef health (Baird *et al.*, 2017; Brodie *et al.*, 2017a; Brodie *et al.*, 2017b), evaluating crown-of-thorns starfish outbreaks (Hock *et al.*, 2014), assessing coral bleaching in the 2016 bleaching event (Baird *et al.*, 2018) and vulnerability to ocean acidification (Mongin *et al.*, 2016). The eReefs marine BGC model has also been used to analyse the feasibility of environmental interventions for delaying coral bleaching onset and reducing resulting coral mortality (Baird *et al.*, 2020a; Harrison, 2024; Scofield *et al.*, 2024). Solar radiation management techniques are proposed for reducing downwelling irradiance and atmospheric options for cooling and shading reefs include fogging and marine cloud brightening (Harrison *et al.*, 2019; Harrison, 2024). Preliminary modelling of cooling and shading interventions indicated an average reduction in incoming solar shortwave radiation of ~6.8% could have reduced bleaching stress by ~50% in the 2015–16 bleaching event and ~65% in the 2016–17 bleaching event, based on a small number of sample reefs modelled in high resolution (Harrison *et al.*, 2019). As reef interventions transition from laboratory and field testing to deployment in the natural environment, numerical modelling will be required to extrapolate performance estimates, optimise deployment strategies and quantify potential risks (Harrison, 2024).

The CBM within the eReefs marine BGC model is a valuable tool for assessing management strategies on the GBR. To widen the number of applications and facilitate model assessment against laboratory experiments, we developed a single-polyp version of the CBM. This single-polyp version can be compared to experiments with varying temperatures, light levels and coral types to assess model skill and determine parameter values such as maximum symbiont growth rates representing different coral species’ temperature ranges and light tolerances.

For the present study, the CBM was configured to simulate a moderate-duration heat (0–4.4 DHW) and light stress laboratory experiment, which is described in Ellis *et al.* (2024). The model configuration was altered to improve the representation of coral species. The time-varying environmental inputs measured in the experiment (irradiance, temperature and nutrient concentrations) were used to force the model. Repeat measurements of experimental photochemical parameters of coral health (maximum quantum yield (*F_v_/F_m_*), the minimum saturating irradiance (*E*_*k*_), the maximum photosynthetic capacity (*r*ETR^MAX^) and the rise of the curve in the light-limited region ($\mathrm{\alpha}$)) were evaluated against model state variables. Specifically, we aimed to assess the skill of the process-based representation in the CBM for simulating experimental bleaching outcomes.

## Materials and Methods

### Model description

The CBM is a mechanistic model of the coral–symbiont relationship that considers temperature-mediated light-driven oxidative stress resulting in symbiont expulsion. The model includes formulations for symbiont growth, pigment synthesis, xanthophyll cycling, reaction centre dynamics and reactive oxygen species (ROS) build-up. The model explicitly represents both the coral host and the symbiont biomass.

The model simulates bleaching when the symbiont cell expulsion rate exceeds zero. This rate depends on ROS concentration (mg O cell^−1^) surpassing a pre-determined bleaching (ROS) threshold of 1.42 × 10^−14^ mg O cell^−1^, below which no bleaching occurs (Suggett *et al.*, 2009).

**Figure 1:**
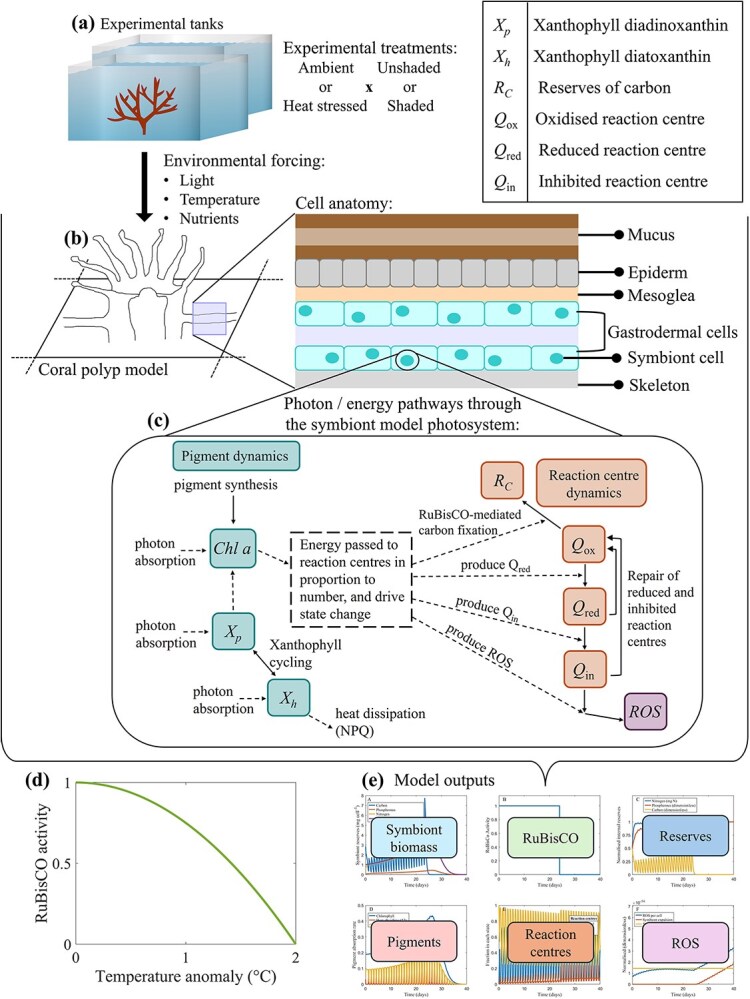
Modelling workflow. Environmental data from a coral heat stress experiment (**a**) input into a single-polyp model (**b**). Photon/energy pathways through the symbiont model photosystem (**c**); the symbiont is contained in two layers of gastrodermal cells. Temperature-dependent RuBisCO activity (**d**) and model outputs (**e**) (modified from Baird *et al.* (2018) and Gustafsson *et al.* (2013)). The coral vector used in this figure is courtesy of Catherine Collier, Great Barrier Reef Marine Park Authority (https://ian.umces.edu/media-library/acropora-spp/). The aquarium tank vector is courtesy of Tracey Saxby, Integration and Application Network, the University of Maryland Center for Environmental Science (https://ian.umces.edu/media-library/aquarium-tank/).

Prior use of the CBM within eReefs involved multi-year simulations on a spatially resolved grid (Baird *et al.*, 2018; Baird *et al.*, 2021). The values for model parameters were calculated from a study using one generic coral type and one Symbiodiniaceae clade (*Symbiodinium* spp.) (Suggett *et al.*, 2008). Hence, a single-polyp configuration of the CBM was developed, driven by the laboratory conditions of light, temperature and nutrient concentrations (Fig. 1b). Continuous with the original configuration, the symbiont cells in the single-polyp model were physically constrained to have a projected area contained in a two-layer gastrodermal cell anatomy (Fig. 1b).

Photon and energy pathways through the symbiont model photosystem are divided into pigment (Fig. 1c, left) and reaction centre dynamics (Fig. 1c, right). Photons absorbed by photosynthetic pigments will change either the internal reserves of carbon, the reaction centre state or the concentration of ROS (Fig. 1c, right). In the symbiont cell, when photons are absorbed and electrons are passed to the reaction centres by the pigments of chlorophyll *a* and diadinoxanthin (*X_p_*), this is termed photochemical quenching (Fig. 1c, left). The pigment diatoxanthin (*X_h_*) absorbs photons and dissipates them as heat as a photoprotective mechanism, termed non-photochemical quenching (NPQ). The xanthophyll cycle is the reversible switching between the pigments diadinoxanthin (photosynthetic) and diatoxanthin (photoprotective). The switching direction is controlled by the fraction of active to inactive reaction centres. Symbiont cells with a large fraction of inhibited reaction centres $({Q}_{in}/{Q}_T>0.5)$ switch from diadinoxanthin (*X_p_*) to diatoxanthin (*X_h_*), and a small fraction of oxidised reaction centres vice versa.

The ribulose-1,5-biphosphate carboxylase/oxygenase (RuBisCO) enzyme catalyses the first step of carbon fixation in the Calvin cycle. The inactivation of RuBisCO-mediated carbon fixation in the model is a temperature-dependent empirical formulation (Eq. 1):


(1) \begin{equation*} {a}_{Q_{\mathrm{ox}}}^{\ast }=\left(1-\exp \left(-\left(2-\Delta T\right)\right)\left)/\right(1-\exp \left(-2\right)\right) \end{equation*}

Temperature anomaly $\left(\Delta T\right)$ was calculated as the difference between the environmental temperature forcing and the MMM. The MMM (28.6°C) is the maximum of the 12 monthly mean SST climatology values for the coral collection site of Jenny Louise Shoal Reef, GBR. The temperature anomaly controls the activity of RuBisCO (${a}^{\ast }_{Q_{\mathrm{ox}}}$); activity varies between inactive at 0 and fully active at 1 (Fig. 1d). For $\Delta T<{0}^{{}^{\circ}}\mathrm{C}$, ${a}_{Q_{\mathrm{ox}}}^{\ast }=1$, and all oxidised reaction centres are available for carbon fixation, and at $\Delta T>{2}^{{}^{\circ}}C$, ${a}_{Q_{\mathrm{ox}}}^{\ast }=0$, and all oxidised reaction centres are unavailable for fixation. Eq. (1) was based on the general reasoning that bleaching stress begins at a temperature anomaly of 1°C. At the NOAA bleaching index threshold of 1°C above climatology, ${a}_{Q_{\mathrm{ox}}}^{\ast }=0.73$ (Baird *et al.*, 2018).

The photosystem state in terms of reaction centres can be oxidised (*Q*_ox_), reduced (*Q*_red_) or inhibited (*Q*_in_). Photons that hit oxidised reaction centres when carbon reserves are depleted and the RuBisCO enzyme is active (${a}_{Q_{\mathrm{ox}}}^{\ast }>0$), lead to increased internal reserves of carbon (carbon fixation). In the case that carbon fixation is inhibited (${a}_{Q_{\mathrm{ox}}}^{\ast }$ = 0, or carbon reserves are replete), a photon encountering an oxidised reaction centre will lead to the oxidised reaction centre becoming reduced (Fig. 1c, right). If a photon hits a reduced reaction centre, then a reduced reaction centre becomes inhibited. In the final state, photons absorbed by inhibited reaction centres generate ROS. The fraction of reaction centres in the state oxidised (${Q}^{*}_{\textrm{ox}} $), reduced (${Q}^{\ast}_{\textrm{red}} $) and inhibited (${Q}^{*}_{\textrm{in}} $) were normalised (0–1, dimensionless) to the total reaction centre concentration (for instance ${Q}^{*}_{ox}\equiv{Q}_{ox}/{Q}_T$).

Inhibited reaction centres can be repaired and returned to an oxidised state. The model includes a repair rate as a function of temperature and assumes that reaction centres would need to repair damage caused by 10 mol photon m^−2^ d^−1^. This light intensity represents the minimum repair rate, below which surface-adapted coral species are impacted by low light (Baird *et al.*, 2018). The term that converts inhibited reaction centres to oxidised reaction centres (Eq. 2):


(2) \begin{equation*} \frac{\partial{Q}_{\mathrm{in}}}{\partial t}=-268\ {m}_{\mathrm{RCII}}{Q}_{\mathrm{in}}=-\frac{\partial{Q}_{\mathrm{ox}}}{\partial t} \end{equation*}

where ${m}_{\mathrm{RCII}}$ is a stoichiometric coefficient for the ratio of reaction centre (RCII) units to photons [mol photon (mol reaction centre)^−1^], and the constant 268 arises from the 10 mol photon m^−2^ d^−1^ limit.

Irradiance levels control the rate of photon absorption. For instance, at reduced light levels, initially, fewer photons will be absorbed by the symbiont’s photosynthetic pigments. After carbon fixation, fewer excess photons are available, resulting in fewer state transitions from oxidised to reduced, reduced to inhibited reaction centres and consequently reduced production of ROS. The stoichiometric ratio of the number of photons that lead to the generation of one ROS (${m}_{P2R}$) was set at 7000 mol photon (mg O_2_)^−1^ in the original model configuration (Baird *et al.*, 2018).

Symbiodiniaceae is characterised by the nitrogen biomass of the cellular structural material and the physiological state by the internal reserves of nitrogen, phosphorous and carbon. The maximum internal reserve that can be reached is assumed to be equal to the biomass of the structural material of the cell. The internal reserves influence symbiont growth, and the mass of these reserves (and the total C:N:P:Chl *a* ratio) depends upon supply and consumption rates. In the symbiont growth dynamics of the model, internal reserves are shared across the symbiont cell population. For symbiont growth, producing an additional cell requires the equivalent of 100% internal reserves of carbon, nitrogen and phosphorous for structural material. Model calculations for symbiont growth are continuous in time for a population. However, we can simplify the impacts of growth on internal reserves using discrete growth, as illustrated in Fig. 3 of Baird *et al.* (2018). In discrete growth from two to three cells, the generation of new structural material from symbiont reserves and the dilution of reserves due to cell division is required. From the two initial cells, two full reserves of nitrogen are reduced to one for the structural material of the new cell, leaving one-third of the remaining nitrogen reserve per cell. The same logic is applied to carbon and phosphorous reserves, reducing phosphorous to one-sixth.

The model accounts for the diffusion-limited supply of the dissolved inorganic nutrients nitrogen and phosphorus. Photon absorption provides fixed carbon to the cell's internal reserves. Nitrogen and phosphorus are directly absorbed into the reserves, whilst carbon is initially fixed through photosynthesis. Carbon, phosphorous and nitrogen reserves of the population are hereafter referred to as symbiont carbon, phosphorous and nitrogen reserves (mg m^−2^). Symbiont nutrient fluxes vary by nutrient uptake from the overlying water column. Internal carbon (${R}_C^{\ast }$), phosphorous (${R}_P^{\ast }$) and nitrogen (${R}_N^{\ast }$) reserves were normalised (0–1, dimensionless) to the maximum of that reserve (for instance ${R}_C^{\ast}\equiv{R}_C/{R}_C^{max}$). These internal reserves are consumed to form structural material at the Redfield ratio. The internal reserves increase when the supply of the nutrient exceeds the consumption for growth and decrease when the consumption for growth exceeds the nutrient supply (Baird *et al.*, 2003).

The concentration of chlorophyll *a*, photosynthetic and photoprotective pigment relates to the symbiont cell population (mg m^−2^). The calculation for the rate of pigment synthesis is based on the incremental benefit of adding pigment to the rate of photosynthesis. The calculation also includes a reduced benefit when carbon reserves are replete, a reduced benefit due to self-shading, and the fraction of inhibited reaction centres (Eq. 12 in Baird *et al.*, 2018).

In the original configuration of the CBM, the ROS detoxification rate ($-(d\left[\mathrm{ROS}]/ dt\right)$_detoxification_) was a temperature-dependent empirical formulation, set at the maximum growth rate of the symbiont cell. Symbiont cells detoxify at the same rate as they grow (Baird *et al.*, 2018). Detoxification was proportional to ROS concentration ([ROS]), allowing [ROS] to reduce to zero (Eq. 3):


(3) \begin{equation*} -\left(d\left[\mathrm{ROS}\right]/ dt\right)_{\mathrm{detoxification}}={\mathrm{\mu}}_{\mathrm{CS}}^{\mathrm{max}}\cdot{R}_N^{\ast}\cdot{R}_C^{\ast}\cdot{R}_P^{\ast}\cdot \left[\mathrm{ROS}\right]\end{equation*}

where ${\mu}_{\mathrm{CS}}^{\mathrm{max}}$ is the maximum growth rate of the symbiont, and ${R}_N^{\ast },{R}_C^{\ast }\ \mathrm{and}\ {R}_P^{\ast }$ are internal reserves of nitrogen, carbon and phosphorous, respectively. This equation for ROS detoxification rate was revised in the new configuration of the model (Eq. 4).

The photophysiological processes of photoadaptation, xanthophyll cycling and reaction centre dynamics represented by the model are quantified into model outputs (Fig. 1e). A complete mathematical description of the CBM can be found in Baird *et al.* (2018).

### Model configuration

The laboratory experiment detailed in this paper enabled us to refine the initial conditions of state variables for the developed version of the CBM (Table 1). A healthy starting state for the experimental study species was achieved by setting the initial symbiont chlorophyll *a* concentration (*Chl*) and symbiont biomass (*CS*) (see Table 1). ROS are generated during normal aerobic cell metabolism, and at low levels are necessary for regulating physiological mechanisms (Weydert and Cullen, 2010). Thus, as ROS naturally occur in cells, the initial ROS concentration was set to half the ROS threshold ($\left[{\mathrm{ROS}}_{threshold}\right]\cdot 0.5$), from previously being set at zero initial ROS (Table 2). An initial ROS concentration of $\left[{\mathrm{ROS}}_{threshold}\right]\cdot 0.5$ reduced the model spin-up time to a stable starting state.

**Table 1:** Initial conditions of state variables used in model configuration

Symbol	Description	Value	Units
***CS***	**Symbiont biomass**	**1**	**mg N m^−2^**
*R_N_*	Reserves of nitrogen	CS*0.5	mg N m^−2^
*R_P_*	Reserves of phosphorous	CS*(1*((1/16)*(30.97/14.01)))*0.5	mg P m^−2^
*R_C_*	Reserves of carbon	CS*((106/16)*(12.01/14.01))*0.5	mg C m^−2^
***Chl***	**Symbiont chlorophyll *a* concentration**	CS*5.6786/30	**mg m^−2^**
*X_p_*	Symbiont diadinoxanthin concentration	Chl*0.2448*0.33	mg m^−2^
*X_h_*	Symbiont diatoxanthin concentration	Chl*0.2448*0.67	mg m^−2^
***Q*_ox_**	**Oxidised reaction centre concentration**	**1.0607 × 10^−7^**	**mg m^−2^**
***Q*_red_**	**Reduced reaction centre concentration**	**7.1695 × 10^−9^**	**mg m^−2^**
***Q*_in_**	**Inhibited reaction centre concentration**	**1.0108 × 10^−7^**	**mg m^−2^**
**[ROS]**	**Reactive oxygen species concentration**	ROSthreshold*0.5	**mg O cell^−1^**

Revised initial conditions are highlighted in bold.

**Table 2:** Parameter and code improvements made to the model (originally Bleach.m) or configuration used to run the model (originally Run_Bleach.m)

Type	Symbol	Source	Description	Baird *et al.* (2018)	Current configuration	Rationale
Parameter	${m}_{P2R}$	Run_Bleach.m	The number of photons that lead to the generation of one ROS	7000 mol photon (mg O_2_)^−1^	3500 mol photon (mg O_2_)^−1^	${m}_{P2R}$ was reduced to simulate a thermally sensitive coral with a less efficient photosynthetic apparatus.
Equation	$-\left(d\left[\mathrm{ROS}\right]/ dt\right)$ _detoxification_	Bleach.m	ROS detoxification	ROS detoxification occurred at any time point	Detoxification commenced when [ROS] > $\left[{\mathrm{ROS}}_{threshold}\right]\cdot 0.5$	If the symbiont cells are full of reserves and thus ‘healthy’, they can effectively detoxify ROS back to half the threshold concentration.
Equation	[ROS]	Bleach.m	ROS concentration	50% diffusion of intracellular ROS concentration to extracellular ROS concentration	All ROS generated contributes to the ROS pool	The term for ROS diffusion was removed due to insufficient evidence tracking the movement of ROS.

A key uncertainty of the model lies in reaction centre dynamics and the ratio of reaction centre II state change to photons. Initial values for the oxidised, reduced and inhibited reaction centre concentration (*Q*_ox_, *Q*_red_ and *Q*_in_, respectively) were obtained from a pre-experimental simulation. The pre-experimental simulation involved running the model forced by ambient experimental data for 3 days. The number of photons that lead to the generation of one ROS (parameter = ${m}_{P2R}$) was set to simulate a thermally sensitive coral species with a less efficient photosynthetic apparatus (Table 2). The model was configured and simulations were run in MATLAB (2023). See Data Availability to access the model code (Baird *et al.*, 2024).

### Model improvements

The comparison with experimental data led us to alter the original detoxification equation (3) (see Table 2). Detoxification ($-(d[\mathrm{ROS}]/ dt)$_detoxification_) was altered to occur only when [ROS] exceeded $\left[{\mathrm{ROS}}_{threshold}\right]\cdot 0.5$ (Eq. 4):


(4) \begin{align*} &-\left( d\left[\mathrm{ROS}\right]/ dt\right)_{\mathrm{detoxification}}=\nonumber\\&\left\{\!\! \! \begin{array}{@{}c@{}}0,\kern0.5em \left[\mathrm{ROS}\right]\le \left[{\mathrm{ROS}}_{threshold}\right]\cdot 0.5\\{}\begin{array}{cc}{\mu}_{\mathrm{CS}}^{\mathrm{max}}\!\cdot\! {R}_N^{\ast}\! \cdot\! {R}_C^{\ast}\!\cdot\! {R}_P^{\ast}\! \cdot\! \left(\left[\mathrm{ROS}\right]\hbox{--} \left[{\mathrm{ROS}}_{threshold}\right]\! \cdot 0.5\right)\! \left( CS/{m}_N\right),\\\left[{\mathrm{ROS}}_{threshold}\right]\cdot 0.5<\left[\mathrm{ROS}\right]\le \left[{\mathrm{ROS}}_{threshold}\right]\end{array}\\{}\begin{array}{cc}{\mu}_{\mathrm{CS}}^{\mathrm{max}}\cdot{R}_N^{\ast}\cdot{R}_C^{\ast}\cdot{R}_P^{\ast}\cdot \left(\left(\left[{\mathrm{ROS}}_{threshold}\right]\cdot 0.5\right)\left( CS/{m}_N\right)\right),\\ \left[\mathrm{ROS}\right]>\left[{\mathrm{ROS}}_{threshold}\right]\end{array}\end{array}\!\! \! \right\} \end{align*}

where ${\mu}_{\mathrm{CS}}^{\mathrm{max}}$ is the maximum growth rate of the symbiont, ${R}_N^{\ast },{R}_C^{\ast }\ \mathrm{and}\ {R}_P^{\ast }$ are internal reserves of nitrogen, carbon and phosphorous, respectively, and $CS/{m}_N$ is the concentration of symbiont cells. When [ROS] is less than half the threshold there is no detoxification (line one of Eq. 4). When [ROS] is greater than half the threshold but less than the threshold (line two and three of Eq. 4), detoxification can bring [ROS] back to half the threshold. Detoxification occurs at a rate of difference between the present [ROS] and the threshold. The rate of detoxification is also dependent on the state of the symbiont cell’s internal energy reserves (${R}_N^{\ast },{R}_C^{\ast }\ \mathrm{and}\ {R}_P^{\ast }$). With a decrease in energy reserves, the rate of detoxification decreases. When [ROS] is greater than the threshold (line four and five of Eq. 4), detoxification occurs at a constant rate and is limited to a maximum of half the threshold, multiplied by the growth rate.

The movement of ROS from the algal symbiont into the coral cytoplasm (Downs *et al.*, 2002), and the leakage of ROS by isolated Symbiodiniaceae cells into surrounding media, have been documented in the past (Tchernov *et al.*, 2004). As the ROS hydrogen peroxide (H_2_O_2_) can cross cell membranes, its release can be measured (Murphy *et al.*, 2022). However, the theory that ROS (specifically H_2_O_2_) leaks from the Symbiodiniaceae cytosol, through the symbiosomal lumen, into the coral host cell is incomplete (Oakley and Davy, 2018) due to a lack of studies that track the production and movement of ROS from Symbiodiniaceae *in hospite* (Dungan *et al.*, 2022). The release of ROS is a balance of ROS production, subsequent removal by the intracellular antioxidant enzyme system and the rate of ROS diffusion across the symbiont cell membrane (Murphy *et al.*, 2022). The term used in Baird *et al.* (2018) to account for ROS diffusion was removed from the new configuration (Table 2); thus, all ROS generated ($\left(d\left[\mathrm{ROS}\right]/ dt\right)$_generation_) contribute to the ROS pool (Eq. 5):


(5) \begin{equation*} \left(d\left[\mathrm{ROS}\right]/ dt\right)_{\mathrm{generation}}=\left(\frac{Q_{\mathrm{in}}}{Q_{\mathrm{T}}}\right)\cdot{k}_I/{m}_{RCII}/{m}_{P2R} \end{equation*}

where ${Q}_{\mathrm{in}}$ and ${Q}_{\mathrm{T}}$ are the oxidised reaction centre concentration and total reaction centre concentration, respectively, ${k}_I$ is the rate of photon absorption, ${m}_{\mathrm{RCII}}$ is the stoichiometric ratio of reaction centre II units to photons and ${m}_{P2R}$ is the stoichiometric ratio of the number of photons that leads to the generation of one ROS.

### Model forcing data

The CBM’s explanatory power for predicting coral bleaching stress under light and temperature treatments when forced by environmental data from the experiment (light, water temperature and nutrient concentrations) was tested. Ellis *et al.* (2024) provides a complete methodology of the experiment from which this data is derived; below we give a brief overview.

**Figure 2:**
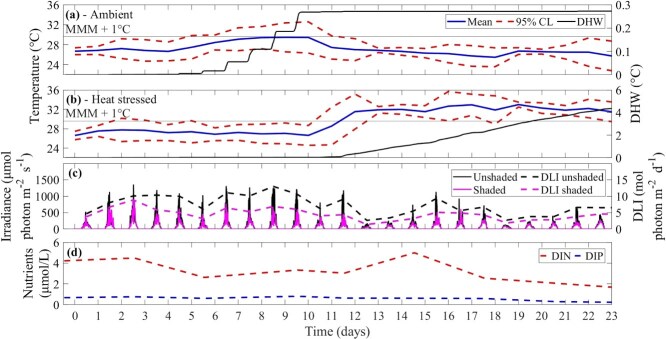
Recorded water temperature (mean temperature (°C) of the day with 95% confidence limits (CL)) and DHW (°C) accumulation for ambient (nominal 26.4°C; **a**) and heat stress (nominal 32.6°C; **b**) temperature treatments, and MMM (28.6°C) + 1°C. Logged PAR irradiance (μmol photon m^−2^ s^−1^) and DLI (mol photon m^−2^ d^−1^) at the depth of coral fragments, in the unshaded and shaded treatment (**c**). Concentration of DIN (μmol/l) and DIP (μmol/l) (**d**) (modified from Ellis *et al.* (2024)).

A manipulative experiment tested the responses of *Acropora divaricata* to an orthogonal combination of shade (two levels: an unshaded control and shaded (30%), maintained continuously) and temperature (two levels: ambient, 26.4°C and heat stress, 32.6°C) over 23 days (Ellis *et al.*, 2024). *Acropora kenti* was also investigated in this experiment but responded negatively to shade application. In this study, we decided to focus on representing *A. divaricata* as the current model configuration cannot depict the behaviour exhibited by *A. kenti,* specifically, it cannot without re-parameterisation predict reduced bleaching stress under increased irradiance conditions combined with heat stress temperatures. The experiment was outdoors under natural lighting at the National Marine Science Centre, Coffs Harbour, Australia (30° 16.062S, 153° 8.244E). Four replicates per treatment equated to 48 experimental tanks, each with a capacity of 600 ml. These tanks were independently supplied with sand-filtered seawater (10 μm) sourced from Charlesworth Bay, Australia (30° 16.028S, 153° 8.356E), at a rate of 100 ml min^−1^. The tanks were placed in a 1200-l water bath. A heat-hold temperature profile (Grottoli *et al.*, 2021) was implemented, featuring a ramp-up period (~0.5°C d^−1^ over 14 days), followed by 9 days of 32.6°C for the heat stress treatment, while the control temperature was set at 26.4°C (Fig. 2). Water temperatures were chosen based on the ambient temperature at Jenny Louise Shoal Reef, GBR (26.4°C; November 2021) and the MMM (28.6°C), plus a temperature anomaly of 4°C.

Photosynthetically active radiation (PAR; μmol photon m^−2^ s^−1^) at the depth of the coral fragments was measured using Odyssey Submersible PAR loggers (5-min interval; cross-calibrated against a LI-COR® LI-250A light meter with an attached LI-192 underwater quantum sensor). The water temperature (°C) of the ambient and heat stress treatment in the experimental tanks was measured with HOBO pendant loggers (HOBO MX-22021, Onset, USA; 15-min interval).

To calculate dissolved inorganic nitrate and phosphate, 20-ml samples of incoming sand-filtered water were collected every 2 days and filtered through polyethersulfone hydrophilic, non-sterile 0.45-$\mathrm{\mu}$m filters (Minisart®) into 30-ml polypropylene vials. Vials were immediately frozen (−18°C) and stored for analysis. Concentrations of nitrate and nitrite (NO_x_), ammonium (NH_4_^+^) and phosphate (PO_4_^3−^) were determined using standard methods 4500-NO_3_^−^ G., 4500-NH_3_ H. and 4500-P, respectively (Eaton *et al.*, 2005), on a flow injection LaChat 8500 (Jeffries *et al.*, 2015).

### Model validation

Model outputs were compared to experimental repeated measures of coral photochemistry. The experiment measured coral photochemistry by pulse amplitude modulated (PAM) fluorometry. Measurements were collected at the beginning (day 0) and every 2 days after. The dark-adapted photochemical efficiency of open reaction centres (maximum quantum yield; *F_v_/F_m_*) was conducted pre-dawn. Rapid light curves (RLCs) on dark-adapted (1 h after sunset) corals evaluated photosynthetic performance and provided saturation characteristics of electron transport. PAM settings were actinic light factor = 1, actinic light intensity = 5, saturation width = 0.8, saturation intensity = 12, signal damping = 2 and gain = 2. The slope of the curve in the light-limited region (alpha, $\mathrm{\alpha}$), the maximum photosynthetic capacity (*r*ETR^MAX^) and the light saturation coefficient (*E*_*k*_) were determined for each fragment. Alpha is proportional to the efficiency of light capture (Schreiber, 2004); alpha indicates the ability of photosystem II to maximise yield before the onset of saturation (Ralph and Gademann, 2005). The intersection of alpha with the maximum photosynthetic rate is the minimum saturating irradiance (*E*_*k*_). The *E*_*k*_ is related to quenching; below the *E*_*k*_, photochemical quenching dominates and above the *E*_*k*_, non-photochemical quenching dominates the fluorescence quenching. *r*ETR^MAX^ is calculated when the electron transport rate (ETR) curve reaches a plateau. A decline in the curve under increased irradiance could be linked to the dynamic downregulation of PSII (White and Critchley, 2004). See Ellis *et al.* (2024) for detailed experimental methods.

Repeated measures analysis of variance (ANOVA) models were fitted to the experimental data to analyse the variation in *F_v_/F_m_*, *E*_*k*_, *r*ETR^MAX^ and alpha with SPSS (version 29.0, SPSS Inc., IBM, USA). For each proxy, a full factorial model was initially fitted with time as a within-subject factor and shade and temperature as between-subject factors. See Ellis *et al.* (2024) for the statistical procedure.

## Results

### Environmental data and experimental results

Detailed experimental results are reported in Ellis *et al.* (2024); key results are summarised here. The mean ambient temperature treatments accumulated 0.27 DHW in the experiment (Fig. 2a). The heat stress temperature treatments accumulated 4.4 DHW (Fig. 2b). The daily ambient PAR irradiance peaked at 818 ± 406 μmol photon m^−2^ s^−1^ (mean ± standard deviation; 10:15 am–12:35 pm; Fig. 2c). From days 10–12, the peak light (unshaded treatment) was 811–1217 μmol photon m^−2^ s^−1^ (daily light integral (DLI) of 6.24–10.66 mol photon m^−2^ d^−1^), which decreased on days 13–14 to 180–252 μmol photon m^−2^ s^−1^ (DLI of 2.64–3.41 mol photon m^−2^ d^−1^) (Fig. 2c). The experimental DLI in the unshaded and shaded treatment was 7.21 ± 2.99 mol photon m^−2^ d^−1^ and 4.48 ± 1.80 mol photon m^−2^ d^−1^, respectively. The dissolved inorganic nitrogen (DIN) and dissolved inorganic phosphorus (DIP) varied with weather conditions as the water was sourced locally from Charlesworth Bay (DIN: 3.35 ± 1.14 μmol/l, DIP: 0.58 ± 0.20 μmol/l; Fig. 2d).

Shading prevented photochemical collapse in *A. divaricata* up to the experiment’s maximum of 4.4 DHW. The photochemical proxies of *F_v_/F_m_, E*_*k*_, *r*ETR^MAX^ and alpha were greater in the shaded treatment than in the unshaded treatment throughout the experiment (*F_v_/F_m_*, *E*_*k*_, and alpha: *P* < 0.05, *r*ETR^MAX^: *P* < 0.01; Table S1). *F_v_/F_m_*, *r*ETR^MAX^ and alpha decreased over time in both temperature treatments (Bonferroni, *P* < 0.01). *E*_*k*_ decreased over time in the heat stress treatment and increased over time in the ambient treatment (Bonferroni, *P* < 0.01). *E*_*k*_ was greater in the ambient than heat stress treatment at later sampling times (*P* < 0.01).

### Simulated bleaching outcomes

Here we describe the model outputs from a new configuration of the CBM. We compare outputs of symbiont growth, pigment concentrations, reaction centre dynamics and symbiont cell expulsion resulting from a build-up of ROS concentration, between the shade treatments at ambient temperature and heat stress temperature. We compare experimental photochemical decline to model-simulated physiological bleaching (symbiont cell expulsion rate).

In the presentation of model results, outputs are presented in the units of milligrams per square metre when they represent a population of cells (for instance symbiont reserves and pigment concentrations), in units of milligrams per cell when they represent the content of an individual cell (for instance ROS concentration) or are dimensionless if normalised (for instance internal reserves normalised to the maximum of that reserve and reaction centre state normalised to total reaction centre concentration). The cell contents are not changed by symbiont cell expulsion (in which a fraction of the population is expelled) but by growth as reserves are consumed to grow.

#### Model behaviour under ambient conditions

**Figure 3:**
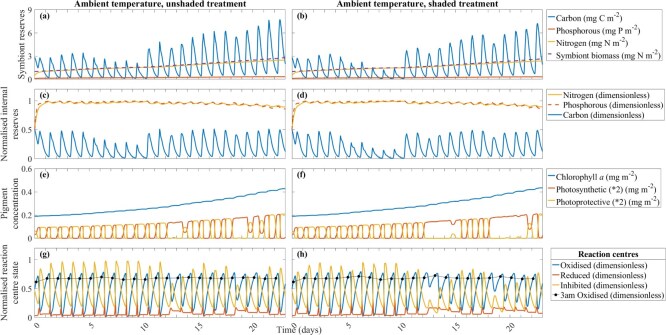
Key model outputs for ambient temperature, unshaded (**a**, **c**, **e**, **g**) and shaded (**b**, **d**, **f**, **h**) treatments: symbiont reserves (carbon, phosphorous and nitrogen (mg m^−2^)) and symbiont biomass (mg N m^−2^) (**a**, **b**), normalised internal reserves (nitrogen, phosphorous and carbon (dimensionless)) (**c**, **d**), the concentration of chlorophyll *a* (mg m^−2^) and photosynthetic and photoprotective xanthophyll pigments (mg m^−2^) (**e**, **f**), and the normalised fraction of reaction centres in the state oxidised (including oxidised state at 3:00 am (black points)), reduced and inhibited (dimensionless) (**g**, **h**). Daily tick marks occur at 0:00 h.

In the ambient temperature treatment, symbiont biomass increased due to growth, and the symbiont population nitrogen reserve was equivalent to this symbiont biomass (Fig. 3a and b). Symbiont carbon reserves displayed an increasing trend from day ~10.5 (Fig. 3a and b) as the RuBisCO enzyme remained active (Fig. S1a). Despite high internal reserves of nitrogen and phosphorous (~1), carbon reserves were limited (<0.5) due to growth exceeding carbon fixation rates (Fig. 3c and d). Internal nitrogen, phosphorous and carbon reserves increased during the day, with nitrogen and phosphorous peaking before solar noon and carbon reserves peaking at solar noon (Fig. 3c and d). Under these conditions, symbiont growth was constrained by the most depleted nutrient, carbon reserves, which, whilst low, were not fully exhausted and were replenished during active RuBisCO activity. Symbiont growth would only cease when an internal reserve was completely depleted, and even when consumption exceeded supply, growth persisted if reserves were available. The model's dynamic nature allowed carbon reserves to fluctuate, consistently remaining <0.5 throughout the simulation, reflecting limitation but not depletion. The faster turnover rate of reaction centres compared to carbon fixation or chlorophyll synthesis enabled carbon reserves to recover during the day despite high consumption.

The model simulated reduced transitions between photosynthetic and photoprotective pigments during periods of low irradiance. In the unshaded treatment, during reduced photon flux from 12–14 and 18–19 days, the xanthophyll pigments were primarily photosynthetic (Fig. 3e). Under 30% shade, the reduced irradiance further delayed these transitions, with the dominance of photosynthetic pigments extending from 12–16 and 18–22 days (Fig. 3f). Reaction centres became inactive during the day and recovered overnight. Consequently, the xanthophyll cycle was primarily photoprotective during the day and shifted to light-absorbing for the early morning.

The varying carbon reserves over a day influenced the fraction of reaction centres in the state oxidised, reduced and inhibited (Fig. 3g and h). At 6:00 am, as cells had a large fraction of oxidised reaction centres, all xanthophyll pigments were photosynthetic. The symbiont and internal carbon reserves were depleted at this time. Throughout the day a greater fraction of reduced reaction centres occurred with a decreased fraction of oxidised reaction centres before greater inhibited reaction centres. At solar noon, as cells had a large fraction of inhibited reaction centres, all xanthophyll pigments were photoprotective (Fig. 3e and f). The inhibited reaction centres were then detoxified overnight into oxidised reaction centres.

**Figure 4:**
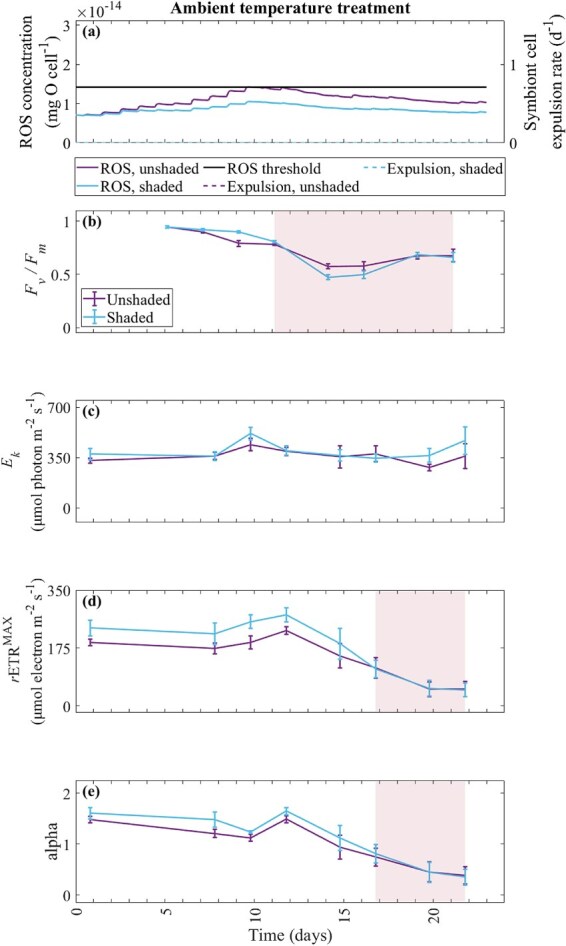
Model-simulated ROS concentration (mg O cell^−1^), symbiont cell expulsion rate (d^−1^) and ROS threshold (1.42 × 10^−14^ mg O cell^−1^) in the ambient temperature treatment for the unshaded and shaded treatments (**a**), in comparison with experimental maximum quantum yield (dimensionless; *F_v_/F_m_*) (**b**), light saturation coefficient (μmol photon m^−2^ s^−1^; *E*_*k*_) (**c**), maximum photosynthetic capacity (μmol electron m^−2^ s^−1^; *r*ETR^MAX^) (**d**) and alpha (dimensionless) (**e**), averaged $\pm$ standard error, recorded for *A. divaricata*. The pink-shaded regions (**b**, **d**, **e**) illustrate a statistical decline in the photochemical proxies from the first sampling event. Daily tick marks occur at 0:00 h.

An increased temperature around solar noon reduced the activity of the RuBisCO enzyme, which reduced carbon fixation and the fraction of oxidised reaction centres. Reduced carbon fixation is demonstrated from 5 to 10 days (Fig. 3c and d). As growth was not restricted to the time of day, the continuous development of symbiont biomass reduced internal carbon reserves. The RuBisCO enzyme never remained inactive for longer than ~2 h over solar noon. At temperatures below the summer climatology maximum, symbionts do not build up toxic levels of ROS, even under the increased irradiance of the unshaded treatment (Fig. 4a). The combination of photoadaptation, carbon fixation and ROS detoxification prevented lethal concentrations of ROS.

#### Ambient temperature, unshaded and shaded treatment.

At ambient temperature, the model simulated increased symbiont biomass and symbiont nitrogen reserves (Fig. 3a and b). Symbiont nitrogen increased ~5-fold (unshaded and shaded treatment); symbiont biomass increased ~3-fold (unshaded and shaded treatment). The unshaded treatment had greater symbiont reserves and symbiont biomass (Fig. 3a) in comparison to the shaded treatment (Fig. 3b) (up to ~18% greater for carbon, ~13% greater for phosphorous, ~14% greater for nitrogen and ~12% greater for biomass). Internal carbon was ~6–8% greater in the unshaded (Fig. 3c) than shaded treatment (Fig. 3d).

In the ambient unshaded treatment, the symbiont cell's photosynthetic capability was not compromised; symbiont biomass increased ~3-fold (Fig. 3a) and the concentration of chlorophyll *a* increased ~2-fold (Fig. 3e).

In the unshaded treatment at ambient temperature, the model-simulated fraction of oxidised reaction centres remained relatively stable over the 23 days (Fig. 3g). The fraction of inhibited reaction centres in the unshaded treatment largely followed the downwelling light irradiance profile of the experiment (Fig. 3g). The highest fraction of reaction centres simulated by the model was in the inhibited state (peak of ~1), followed by oxidised (peak of ~0.5–1) and reduced (peak of ~0.2–0.3) (Fig. 3g). The fraction of reduced and oxidised reaction centres was greater in the shaded treatment than in the unshaded treatment (~18–27% greater for reduced and ~5–19% greater for oxidised reaction centres) (Fig. 3h). The shaded treatment had ~8–24% less inhibited reaction centres than the unshaded treatment. In the shaded treatment, a reduced fraction of inhibited reaction centres (on days 12 and 18) and an increased fraction of oxidised reaction centres at 3:00 am (on days 13 and 19) (Fig. 3h) coincided with a xanthophyll cycle that was primarily photosynthetic (Fig. 3f).

Symbiont cell expulsion was not simulated by the model at ambient temperature (Fig. 4a). In the unshaded treatment, ROS concentration increased to the ROS threshold at ~9.5–12 days but never exceeded the threshold. After 12 days, ROS concentration slowly declined to 1.03 × 10^−14^ mg O cell^−1^ at the end of the experimental timeframe. ROS concentration was diluted by increased symbiont biomass in both shade treatments. Peak ROS concentration was reduced by ~0.3–0.5 × 10^−14^ mg O cell^−1^ in the shaded treatment compared to the unshaded treatment. Whilst no bleaching was simulated by the model at ambient temperature, photochemical adjustments were observed for this treatment in the experiment (Fig. 4b–e). At ambient temperature, *F_v_/F_m_* decreased from 5 to 11–21 days (Bonferroni, *P* < 0.01) and *r*ETR^MAX^ and alpha decreased from 0 to 16–21 days (Bonferroni, *P* < 0.01). Fluctuations in the ambient temperature above the MMM occurred before 12 days, accumulating 0.27 DHW. As the ambient temperature fluctuated above the MMM, *E*_*k*_ was greater in the heat stress treatment during early sampling times (*P* < 0.01). Coinciding with these experimental temperature fluctuations, the experimental light was logged at its highest irradiance.

#### Heat stress temperature, unshaded and shaded treatment

**Figure 5:**
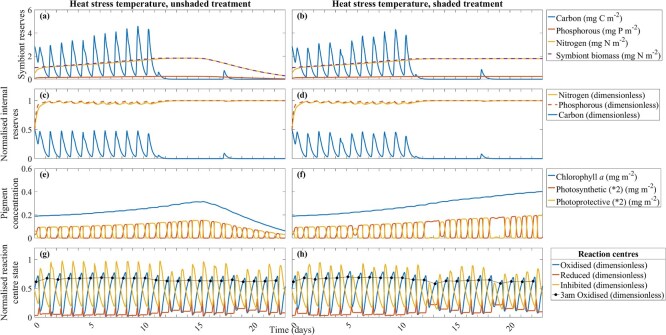
Key model outputs for the heat stress temperature, unshaded (**a**, **c**, **e**, **g**) and shaded (**b**, **d**, **f**, **h**) treatments: symbiont reserves (carbon, phosphorous and nitrogen (mg m^−*2*^)) and symbiont biomass (mg N m^−*2*^) (**a**, **b**), normalised internal reserves (nitrogen, phosphorous and carbon (dimensionless)) (**c**, **d**), the concentration of chlorophyll *a* (mg m^−*2*^) and photosynthetic and photoprotective xanthophyll pigments (mg m^−*2*^) (**e**, **f**), and the normalised fraction of reaction centres in the state oxidised (including oxidised state at 3:00 am (black points)), reduced and inhibited (dimensionless) (**g**, **h**). Daily tick marks occur at 0:00 h.

In the heat stressed unshaded treatment, symbiont biomass increased 1.8-fold from 1 to 14.5 days (Fig. 5a). When the temperature increased at 11 days, the RuBisCO enzyme became inactive (Fig. S1b), and carbon fixation ceased. Symbiont biomass declined from ~14.5 days in the unshaded treatment due to symbiont cell expulsion (Fig. 5a). From ~12.5 days, the shaded treatment at heat stress temperature stabilised symbiont biomass and symbiont reserves of nitrogen at ~2 mg N m^−2^ (Fig. 5b).

In the heat stressed unshaded treatment, growth in symbiont biomass was strongly carbon-limited. Preceding symbiont cell expulsion, internal carbon reserves exhibited diel variation at 0–10 days (Fig. 5c). At ~12.5 days, symbiont carbon (0.01 mg C m^−2^; shaded and unshaded treatment) (Fig. 5a and b) and internal carbon reserves (unshaded: 0.1% of maximum carbon reserve, shaded: 0.05% of maximum carbon reserve) (Fig. 5c and d) became limited.

Under increased temperatures, RuBisCO-mediated carbon fixation was inhibited, leading to increased excess photons and more state transitions of reaction centres from oxidised to reduced to inhibited. Conversely, under reduced temperatures, RuBisCO-mediated carbon fixation was not inhibited, and so fewer excess photons resulted in fewer state transitions of reaction centres. For the time-varying activity of RuBisCO, see Fig. S1b. At ~17–18 days in the heat stress treatment, a peak in RuBisCO activity of 0.63 (dimensionless) coincided with a peak in symbiont carbon (~0.8 and ~0.9 mg C m^−2^, for the unshaded and shaded treatment, respectively; Fig. 5a and b), and a peak in internal carbon reserves (unshaded: 9.7% of maximum carbon reserve, shaded: 8.5% of maximum carbon reserve; Fig. 5c and d), and a reduction in the fraction of inhibited reaction centres (unshaded: 33% of the total reaction centre concentration, shaded: 42% of the total reaction centre concentration; Fig. 5g and h).

Increased internal nitrogen (96–97% of maximum nitrogen reserve) and phosphorous reserves (98% of maximum phosphorous reserve) (Fig. 5c) were forced by high forcings of DIN and DIP (Fig. 2d). At heat stress temperature, the internal nitrogen and phosphorous reserves ceased to fluctuate at ~12.5 days (Fig. 5c and d); the reserves were stable for the remaining timeframe (~100% of maximum reserves). The application of shade at heat stress temperature did not markedly alter the internal nitrogen and phosphorous reserves (Fig. 5d) from the unshaded treatment (Fig. 5c). Internal carbon reserves were only ~1–11% reduced in the shaded than unshaded treatment.

In the heat stressed unshaded treatment, the xanthophyll cycle transitioned rapidly between photosynthetic and photoprotective (Fig. 5e). The concentration of xanthophyll pigments (photosynthetic diadinoxanthin and photoprotective diatoxanthin) and chlorophyll *a* decreased ~1 day after symbiont cell expulsion commenced. Whereas, in the shaded treatment at heat stress temperature, the concentration of xanthophyll pigments and chlorophyll *a* continued to increase until the end of the experiment (Fig. 5f). The concentration of chlorophyll *a* increased by ~0.2 mg m^−2^ from 0 to 22 days.

In the heat stressed unshaded treatment, the average fraction of inhibited reaction centres (54% of the total reaction centre concentration) was greater than oxidised (38% of the total reaction centre concentration) or reduced (8% of the total reaction centre concentration) reaction centres (Fig. 5g). A high fraction of inhibited reaction centres indicated photons absorbed by reaction centres led to the generation of ROS. In the shaded treatment, the fraction of inhibited reaction centres was reduced by ~7–19% (Fig. 5h), compared to the unshaded treatment. The fraction of oxidised reaction centres at 3:00 am declined on days 13 and 19 (Fig. 5h), coinciding with a photosynthetic xanthophyll cycle (Fig. 5f).

**Figure 6:**
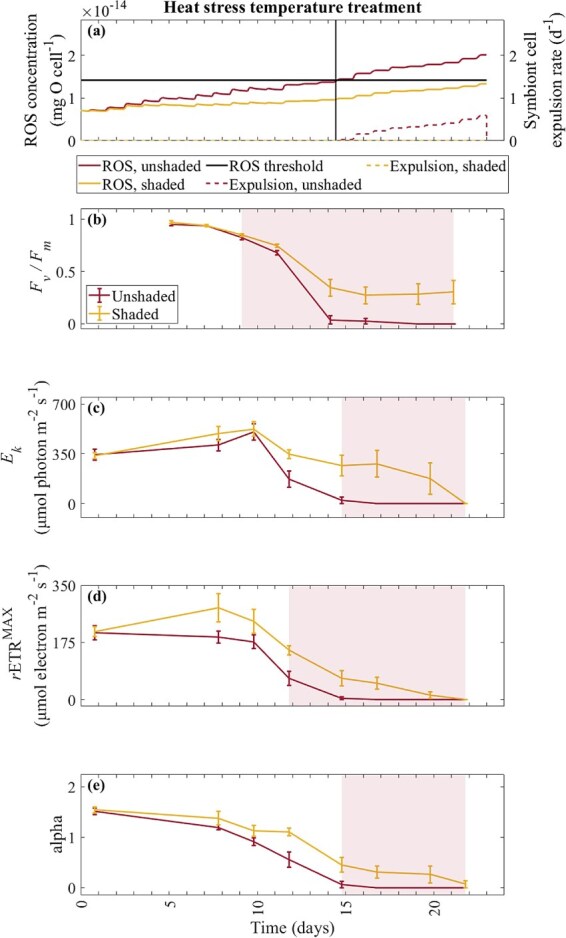
Model-simulated ROS concentration (mg O cell^−1^), symbiont cell expulsion rate (d^−1^) and ROS threshold (1.42 × 10^−14^ mg O cell^−1^) in the heat stress temperature treatment for the unshaded and shaded treatments (**a**), in comparison with experimental maximum quantum yield (dimensionless; *F_v_/F_m_*) (**b**), light saturation coefficient (μmol photon m^−2^ s^−1^; *E*_*k*_) (**c**), maximum photosynthetic capacity (μmol electron m^−2^ s^−1^; *r*ETR^MAX^) (**d**) and alpha (dimensionless) (**e**), averaged $\pm$ standard error, recorded for *A. divaricata*. The black vertical line represents the model-simulated onset of bleaching (**a**). The pink-shaded regions (**b**, **c**, **d**, **e**) illustrate a statistical decline in the photochemical proxies from the first sampling event. Daily tick marks occur at 0:00 h.

The model-simulated onset of bleaching in the heat stress and unshaded treatment started at ~14.5 days when ROS concentration exceeded the ROS threshold (Fig. 6a), coinciding with an inactive RuBisCO enzyme and depleted carbon reserves. By day 14.5, the heat stress treatment remained above the MMM, coinciding with zero RuBisCO activity.

The model-simulated onset of bleaching in the heat stress and unshaded treatment coincided with a significant decline in the experimental photochemical proxies, as indicated by the shaded region (Fig. 6b–e). The onset of bleaching for the unshaded treatment was simulated by the model 5.5 days after a significant decline in the experimental *F_v_/F_m_*. Experimental *F_v_/F_m_* showed increased bleaching stress with DHW accumulated over time, similarly, as simulated by the model. Experimental *F_v_/F_m_* in the heat stress treatment decreased from 5 to 9–21 days (Bonferroni, *P* < 0.01) (Fig. 6b). The model-simulated onset of bleaching was more closely matched with a significant decline in experimental *r*ETR^MAX^ (difference of 2.5 days) and *E*_*k*_ and alpha (difference of 0.5 days). *r*ETR^MAX^ decreased from 0 to 12–21 days (Bonferroni, *P* < 0.01) (Fig. 6d). *E*_*k*_ (Fig. 6c) and alpha (Fig. 6e) decreased from 0 to 14–21 days (Bonferroni, *P* < 0.01).

In the shaded treatment, ROS concentration increased to 1.34 × 10^−14^ mg O cell^−1^ by day 23, but as this was below the ROS threshold (1.42 × 10^−14^ mg O cell^−1^), the symbiont cell expulsion rate was zero in the heat stress and shaded treatment (Fig. 6a). Shade prevented photochemical collapse in *A. divaricata*. However, even with shade application, a significant photochemical decline was observed in the experiment. The photochemical proxies of *F_v_/F_m_*, *E*_*k*_, *r*ETR^MAX^ and alpha measured for *A. divaricata* decreased significantly with DHW accumulation in the heat stress treatment (Fig. 6b–e).

## Discussion

### Comparing experimental to model-simulated coral bleaching

In the unshaded treatment, the model captured the observed coral bleaching under heat stress conditions. This suggests that the photophysiological processes represented by the model, including photoadaptation, xanthophyll cycle dynamics and reaction centre state transitions, were influential in representing the observed coral bleaching under these environmental conditions. Furthermore, the model-simulated onset of bleaching matched closely with an initial photochemical decline as observed in the experiment. This is significant as the model not only simulated the observed pattern of bleaching under heat stress conditions, in the unshaded treatment, but it also captured the timing and progression of the bleaching process, which further supports the model’s mechanistic processes.

The model simulated that high light coupled with increased temperature leads to inhibited reaction centres and accumulated ROS. The model-simulated changes in ROS concentration aligned with experimental photochemical measurements. This provides a powerful validation of the temperature-mediated, light-driven mechanism of bleaching used by the model. The model’s ability to capture bleaching in the treatment of the greatest environmental stress and its relationship to underlying physiological processes observed *ex situ* emphasises the potential of this modelling tool for predicting reef futures.

The model simulated reduced health but no symbiont cell expulsion for the shaded treatment at heat stress temperature. Model-simulated photochemical changes in the shaded treatment at heat stress temperature were insufficient to cause bleaching; ROS concentration gradually increased up to the threshold by the end of the experiment but never exceeded it. Symbiont cell expulsion was simulated by the model only in the unshaded treatment at heat stress temperature. In the experiment, shade alleviated experimental photochemical stress under heat stress; *F_v_/F_m_* did not decrease to zero and the decline in *E*_*k*_, *r*ETR^MAX^ and alpha was slowed, as opposed to the unshaded treatment. Previous research has demonstrated that shading reduces coral bleaching risk (Butcherine *et al.*, 2023), which supports using shade-based management interventions to reduce coral bleaching stress and coral mortality (Harrison, 2018). However, even with the application of shade, a significant decline in coral health was observed in our experiment due to the accumulation of heat stress. The temperature treatments may have played a larger role in the experimental bleaching than could be simulated by the model due to unaccounted factors, for instance, pre-experimental stress, acclimatisation or symbiont shuffling.

### Evaluating model improvements

The decision to remove the coefficient for ROS diffusion, increase the initial concentration of ROS within a cell and reduce the number of photons per ROS in this configuration of the model would have further increased the model-simulated ROS concentration. Other changes to initial conditions are not discussed as the model’s photochemistry was shown to adjust within 2 days. An increased ROS concentration increased the symbiont cell expulsion rate. Experimental evidence of ROS movement from the symbiont to the coral host would confirm the relevance of using a ROS diffusion term in the CBM. Further model validation would be required to match simulated changes in photochemistry to observed photochemical proxies.

Revising a parameter that influences the sensitivity to light may determine the degree of bleaching simulated by the model. The build-up of ROS simulated by the model is both temperature-mediated and light-driven. The number of photons that lead to the generation of one ROS was reduced to represent the heat-sensitive reef-building coral of the experimental study—*A. divaricata*. If the number of photons per ROS was not reduced, the model would simulate less ROS concentration in the shaded treatment at heat stress temperature and a reduced symbiont cell expulsion rate in the unshaded treatment at heat stress temperature. Further reducing the number of photons per ROS would increase the bleaching stress simulated by the model in the shaded treatment at heat stress temperature. The stoichiometric nature of the parameter for the number of photons per ROS suggests it might be common across symbiont species.

The improvements made to the model contributed to the fit between the model-simulated and observed bleaching. This improved match was due to the model’s more accurate representation of the mechanism through which temperature-mediated light-driven oxidative stress leads to ROS concentration build-up, and consequently symbiont cell expulsion. The model changes outlined above increased the bleaching stress and subsequent bleaching simulated by the model. An increased ROS concentration from the model changes meant that the onset of symbiont cell expulsion under heat stress conditions, in the unshaded treatment, closely corresponded to the observed photochemical decline in *A. divaricata*. Specifically, the model-simulated bleaching commenced 0.5 days after *E*_*k*_ and alpha, 2.5 days after *r*ETR^MAX^ and 5.5 days after *F_v_/F_m_*, as observed in the experiment. Without these model improvements, we would not expect such a strong degree of model fit to the observed bleaching.

### Model configuration limitations

Nutrient oversupply had no impact on the model outputs. The model simulated a nutrient-replete system with high normalised internal reserves of nitrogen and phosphorous, independent of temperature or shade treatment. The nutrient-replete system simulated by the model and forced by the experimental environmental data may not fully represent the natural coral environment. Many coral reefs are in oligotrophic tropical waters with undetectable levels of DIN (<1 μmol l^−1^) and DIP (<0.1 μmol l^−1^) (Tanaka *et al.*, 2007). The averaged experimental DIN (1.68 μmol l^−1^) was outside this range, and the averaged experimental DIP (0.19 μmol l^−1^) was also higher but was less than what is considered intermediate nutrient concentrations (DIP < 0.35 μmol l^−1^) found in upwelling regions or coastal areas impacted by riverine and groundwater discharge (Tanaka *et al.*, 2007). Chronic nutrient exposure increases the prevalence of disease and severity of coral bleaching (Vega Thurber *et al.*, 2014). An increased susceptibility to temperature- and light-induced bleaching has been linked with increased levels of DIN in combination with limited phosphate concentrations (Wiedenmann *et al.*, 2013). Specifically, nutrient exposure may increase symbiont abundance (Marubini and Davies, 1996), thus increasing coral bleaching susceptibility during heat stress (Wooldridge, 2009; Cunning and Baker, 2013; Wiedenmann *et al.*, 2013). In the model, symbiont cells are expelled due to symbiont ROS levels. However, increased activities of CAT and SOD in response to increased concentrations of the ROS H_2_O_2_ at elevated seawater temperature have been found in both coral tissue and zooxanthellae (Higuchi *et al.*, 2008). This is most likely since H_2_O_2_ is a diffusive ROS molecule that readily diffuses across biological membranes and so is not restricted to its point of synthesis (Lesser, 2006; Baird *et al.*, 2009). The potential impact of host–ROS concentration is not currently included in the model.

As the model could not incorporate nutrient oversupply into predictions, this may have caused a mismatch between the observed and model-simulated coral bleaching. Considering the above-mentioned impacts linked with increased nitrogen levels, we could expect the model to simulate increased bleaching stress if nutrient oversupply was introduced. With increased bleaching stress, an earlier onset of bleaching may have been simulated by the model, and perhaps increased rates of symbiont cell expulsion.

Demonstrating the effects of nutrient oversupply involves representing complex interactions and processes in the coral reef environment. The current simplification of formulations used by the model allows a comprehensive set of processes to be represented, from nutrient and photochemical interactions to coral symbiosis (Baird *et al.*, 2018). Further tweaking of the model configuration may be required to best represent dynamics at the lower nutrient levels typical of most coral reef environments; this is yet to be tested.

In addition to elevated light and heat stress, corals bleach in response to any stressor that disrupts photochemical quenching, namely high levels of dissolved carbon dioxide, salinity extremes, environmental contaminants, low light and cold stress. Dissolved oxygen, pH and salinity are commonly monitored environmental variables in a coral bleaching study and were measured during the experiment to ensure their variation did not confound bleaching results. In the model, these variables do not drive growth rate or photosystem efficiency. Including these variables in growth equations and as input forcings, thus representing them as potential stressors, may provide a more accurate projection of coral bleaching. This would enable the representation of different bleaching events, for instance capturing the localised freshwater bleaching that occurred in 2008–09 and 2010–11 from high freshwater discharges (Lough *et al.*, 2015).

As acknowledged in Baird *et al.* (2018), not all processes in the CBM are mechanistic. The inactivation of RuBisCO-mediated carbon fixation, the repair rate of inhibited reaction centres and the detoxification rate of ROS are temperature-dependent empirical formulations. The equation for ROS detoxification is an empirical formulation based on observation rather than mechanistic understanding. ROS detoxification is directly proportional to the maximum growth rate of the symbiont as a simplification—if healthier, faster growing cells have more resources for detoxification. A quantitative understanding of the underlying biochemical reactions is necessary to formulate more mechanistic process descriptions in the CBM.

The model simplifies the carbon fixation process and does not fully capture how excess energy is managed by the symbiont when carbon reserves are full. The model assumes that when carbon reserves are full or the RuBisCO enzyme is inactive, photons are not used in carbon fixation. Instead, they lead to the reduction of an oxidised reaction centre. This simplification prioritises simulating reaction centre dynamics and ROS production as drivers of coral bleaching. Whilst the model does not directly simulate the release of carbon, if the host cannot use translocated photosynthate from the symbiont, the model assumes it is released as mucus. The model does not explicitly represent the storage of fixed carbon as starch, instead, it uses generic ‘reserves of carbon’. Future iterations of the model should aim to incorporate more detailed mechanisms of carbon storage and release in symbiotic algae.

### Future model advancement

Future CBM evaluation should compare model-simulated symbiont cell expulsion to observed symbiont cell expulsion and model-simulated photochemistry to observed photochemistry. In this paper, we compared photochemical decline to symbiont cell expulsion rate. The build-up of ROS in Symbiodiniaceae is a physiological response to environmental stress. Comparing the model-simulated physiological response to the observed physiological response would be the most suitable way to validate the mechanism of ROS-induced bleaching used by the model. Comparing the model-simulated photochemical response to the observed photochemical response would validate the photosystem represented by the model. This represents an avenue for future model evaluation.

Further information is required to resolve the role of ROS within the coral holobiont and to determine the sequence of events that lead to coral bleaching. This will determine whether a model based on ROS-induced bleaching is the best way to represent the coral bleaching process. Previous studies have detected decreased photosynthetic efficiency and increased oxidative stress in thermally stressed corals (Lesser, 1997; Downs *et al.*, 2002; Saragosti *et al.*, 2010). Increased concentrations of ROS during acute thermal stress are a product of photochemical stress rather than a proximate cause of stress. Thus, coral bleaching may be driven by ROS-independent mechanisms (Morris *et al.*, 2019; Rädecker *et al.*, 2021; Rädecker *et al.*, 2023). Further coral bleaching studies are required to isolate the mechanisms of bleaching.

ROS levels associated with coral bleaching could be inferred through other proxies. Due to the difficulty with measuring ROS concentration (it is often measured as a fluorescent signal rather than a mass per unit volume) and the limitations presented with current methodologies (Murphy *et al.*, 2022), there is no precise value for ROS toxicity in the literature (Scofield *et al.*, 2024). An indirect comparison to model-simulated ROS levels could be made with antioxidant enzyme analysis. Components of the antioxidant system, namely superoxide dismutase, catalase and glutathione peroxidase enzymes, are responsible for converting intracellular accumulations of ROS into water before levels supersede a concentration threshold, thus avoiding significant oxidative damage (Baird *et al.*, 2009). Elevated antioxidant enzyme activity indicates increased levels of ROS (Lesser *et al.*, 1990). Future evaluation of the CBM could consider using antioxidant enzyme analysis to infer ROS-induced levels of stress in coral species.

## Conclusions

This study validates the model representation of the photophysiological processes of the coral–symbiont relationship and temperature-mediated, light-driven bleaching impacts. The model captured the observed coral bleaching under heat stress conditions in the unshaded treatment. The model-simulated timing for the onset of bleaching closely corresponded (within a few days) with a significant photochemical decline in the experiment. For the heat stressed but shaded experimental treatment, the model simulated reduced stress that did not exceed the bleaching threshold, whilst in the experiment this treatment experienced reduced and delayed photochemical decline relative to the unshaded heat stressed treatment. This indicates that the model simulated greater protection from bleaching impacts under reduced light levels than in the experiment. Likewise, increased temperatures may have negated some benefits of shade in the experiment.

Further validation of the CBM should be conducted in various environmental scenarios and scaled up from a short-duration heat stress experiment to a more realistic representation of an MHW event. Future model evaluation could also consider comparing model-simulated symbiont cell expulsion to observed symbiont cell expulsion and model-simulated photochemistry to observed photochemistry. Alternatively, quantifying the activity of antioxidant enzymes in corals may provide a more suitable comparison to the model’s simulation of ROS-induced bleaching. Changes to the model configuration could consider representing lower nutrient levels typical of most coral reef environments and replacing empirical-based formulations with more mechanistic process descriptions.

From this initial model evaluation, we demonstrate the utility of numerical modelling in predicting bleaching outcomes under the multiple stressors of heat and light. We anticipate that process-based mechanistic modelling can provide a much needed assessment of reef interventions, management strategies and predictions of coral bleaching under various climate scenarios. Thus, the CBM has many potential applications, and we envision this study as a foundation for its continued development.

## Supplementary Material

Web_Material_coaf020

## Data Availability

The experimental data underlying this article will be shared following reasonable request to the corresponding author. The code to run the CBM (Bleach_SCU_lab_exp_2024.m and Run_Bleach_SCU_lab_exp_2024.m) is available on GitHub at https://github.com/gbrrestoration/coral-bleaching-model, in the ‘main’ branch of the ‘coral-bleaching-model’ repository (Baird *et al.*, 2024).
