# Supplementary material for: A photophysiological model of coral bleaching under light and temperature stress: experimental assessment
Source: Conserv Physiol. 2025 Apr 15;13(1):coaf020. doi: 10.1093/conphys/coaf020 (PMC11997550; doi:10.1093/conphys/coaf020)
Supplement: Web_Material_coaf020 [file web_material_coaf020.zip › supplementary materials.pdf]

A photophysiological model of coral bleaching under light and temperature stress: experimental assessment

Sophia L. Ellis*[1], Mark E. Baird[2], Luke P. Harrison[3], Kai G. Schulz[4], and Daniel P. Harrison[1,5]

Affiliations

[1] *National Marine Science Centre, School of Environment, Science and Engineering, Southern Cross University, Coffs Harbour, NSW 2450, Australia*

[2] *Environment Research Unit, Commonwealth Scientific and Industrial Research Organisation, Hobart, TAS 7001, Australia*

[3] *School of Aerospace, Mechanical and Mechatronic Engineering, University of Sydney, Sydney, NSW 2006, Australia*

[4] *Centre for Coastal Biogeochemistry, School of Environment, Science and Engineering, Southern Cross University, Lismore, NSW 2480, Australia*

[5] *School of Geosciences, University of Sydney, Sydney, NSW 2050, Australia*

Supplementary materials

Table S1: Repeated measures ANOVA results for experimental photochemical proxies of F_v / F_m (dimensionless), E_k (µmol photon m^{-2} s^{-1}), rETR^{MAX} (µmol electron m^{-2} s^{-1}), and alpha (dimensionless), for *Acropora divaricata*. For each parameter, a full factorial model was initially fitted with time as a within-subject factor and shade and temperature as between-subject factors. Values in bold represent a significant difference (P < 0.05) between treatment and control.

**Proxy**		**Shade**	**Temp**	**Time x temp**	**Time x shade**	**Temp x shade**	**Time x temp x shade**
F_v / F_m	*F*	5.11	49.19	26.96	0.65	4.31	3.35
	df	1, 27	1, 27	1.73, 27	1.73, 27	1, 27	1.93, 27
	P	**0.04**	**< 0.01**	**< 0.01**	0.51	0.06	0.05
E_k	*F*	6.80	8.71	4.78	0.64	1.93	1.47
	df	1, 27	1, 27	4.16, 27	4.16, 27	1, 27	4.13, 27
	P	**0.01**	**< 0.01**	**< 0.01**	0.64	0.17	0.21
rETR^{MAX}	*F*	12.23	26.90	8.67	1.58	0.62	0.48
	df	1, 27	1, 27	4.06, 27	4.06, 27	1, 27	4.02, 27
	P	**< 0.01**	**<0.01**	**< 0.01**	0.18	0.44	0.75
alpha	*F*	5.82	29.01	6.91	1.05	1.22	0.58
	df	1, 27	1, 27	3.63, 27	3.63, 27	1, 27	3.58, 27
	P	**0.02**	**< 0.01**	**< 0.01**	0.38	0.28	0.66

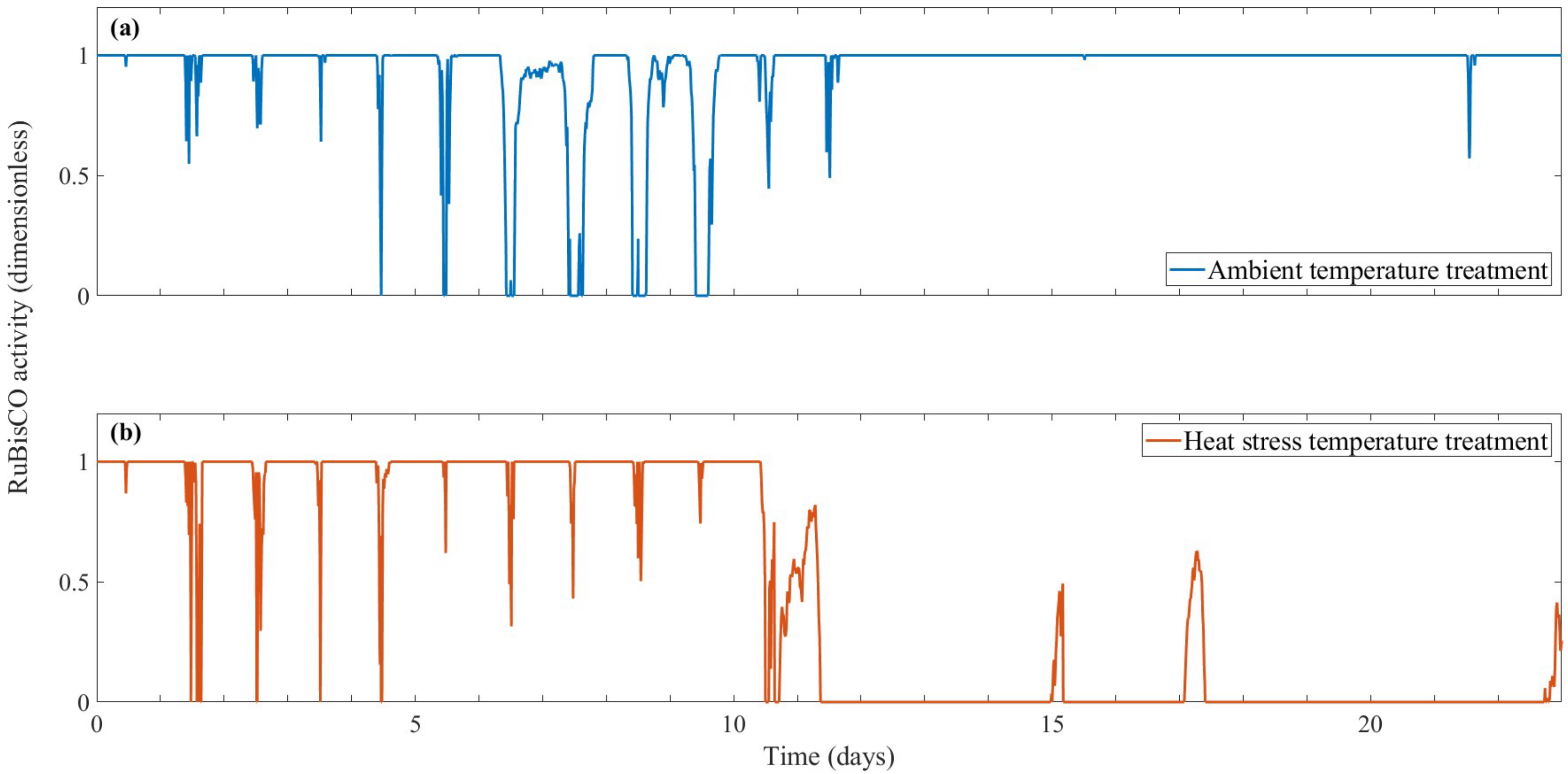

Fig. S1: Simulated RuBisCO activity (dimensionless) for the ambient temperature, unshaded treatment (a) and the heat stress temperature, unshaded treatment (b). Daily tick marks occur at 0:00 h.
